# 69 Methamphetamine Positivity Prior to Burn Surgery Does not Adversely Affect Intraoperative or Inpatient Outcomes

**DOI:** 10.1093/jbcr/irad045.043

**Published:** 2023-05-15

**Authors:** Clifford Sheckter, Nada Rizk, David Crawford, Yvonne Karanas, Christopher Barnes, Tam Pham

**Affiliations:** Stanford University, Palo Alto, California; Stanford University, Palo Alto, California; University of Washington, Seattle, Washington; Santa Clara Valley Medical Center, Stanford, California; University of Washington, Seattle, Washington; University of Washington, Seattle, Washington; Stanford University, Palo Alto, California; Stanford University, Palo Alto, California; University of Washington, Seattle, Washington; Santa Clara Valley Medical Center, Stanford, California; University of Washington, Seattle, Washington; University of Washington, Seattle, Washington; Stanford University, Palo Alto, California; Stanford University, Palo Alto, California; University of Washington, Seattle, Washington; Santa Clara Valley Medical Center, Stanford, California; University of Washington, Seattle, Washington; University of Washington, Seattle, Washington; Stanford University, Palo Alto, California; Stanford University, Palo Alto, California; University of Washington, Seattle, Washington; Santa Clara Valley Medical Center, Stanford, California; University of Washington, Seattle, Washington; University of Washington, Seattle, Washington; Stanford University, Palo Alto, California; Stanford University, Palo Alto, California; University of Washington, Seattle, Washington; Santa Clara Valley Medical Center, Stanford, California; University of Washington, Seattle, Washington; University of Washington, Seattle, Washington

## Abstract

**Introduction:**

The treatment of burn patients using amphetamines is challenging for hemodynamic and behavioral reasons. In addition, recent amphetamine use may also pose intraoperative risks. Due to insufficient literature to guide the safety and timing of operative care, wide practice variation exists in the operative timing for this patient population. We hypothesize that burn excision without negative amphetamine toxicology is safe.

**Methods:**

We queried the operative and electronic health records of amphetamine-positive patients treated from 2017-2022 at two ABA verified burn centers. The practice guideline at Center A obtains admission toxicology only and proceeds with surgery based on hemodynamic status and operative urgency, whereas Center B sends daily toxicology until a negative test results. The primary safety outcome was the use of vasoactive agents during the index operation. This risk was modeled via logistic regression and adjusted for injury severity and hospital day for index operation. Secondary outcomes included death and inpatient complications.

**Results:**

A total of 270 patients were identified during the study period. Mean age was 45.5 years (SD 12.9 years), median TBSA was 9.2% and the majority were male (76.7%). There were no significant differences in injury size, age, or male/female proportion between the two sites. Whereas Center A only tested once on admission, Center B obtained a median of 4 toxicology screens prior to surgery. There were no difference in mean induction systolic blood pressure between the 2 sites, although Center A had more patients who had SBP< 95 or >160mmHg on induction (one-way ANOVA p=0.596). The adjusted OR of requiring vasoactive support during surgery was not associated with negative toxicology status (p=0.821). A larger injury size (%TBSA >20) conferred a significant higher risk of vasoactive support (adj. OR 13.42 [3.90 – 46.23], p< 0.001). Clinical outcomes at the 2 sites were similar in the terms of mortality, number of operations, stroke, and hospital length of stay.

**Conclusions:**

Comparison between 2 ABA verified burn centers identifies a significant variation in practice with regards to serial amphetamine screenings prior to burn surgery. This study indicates that negative toxicology does not impact intraoperative anesthesia management nor subsequent burn clinical outcomes.

**Applicability of Research to Practice:**

Serial toxicology tests are unnecessary to guide operative timing of burn patients with amphetamine use.

